# The complete mitochondrial genome of *Amblyomma geoemydae* (Ixodida: Ixodidae)

**DOI:** 10.1080/23802359.2019.1640643

**Published:** 2019-07-18

**Authors:** Qiao-Cheng Chang, Yang Hu, Teng-Cheng Que, Yun-Xi Liu, Jin-Guo Zhu, Pei-Wen Diao

**Affiliations:** a College of Animal Science and Veterinary Medicine, Heilongjiang Bayi Agricultural University, Daqing, P.R. China;; b Guangxi Zhuang Autonomous Region Terrestrial Wildlife Medical-aid and Monitoring Epidemic Diseases Research Center, Nanning, P.R. China;; c Department of Infection Management and Disease Control, Chinese PLA General Hospital, Beijing, P.R. China;; d Manzhouli Customs District, Manzhouli, P.R. China

**Keywords:** *Amblyomma geoemydae*, mitochondrial genome, phylogenetic analysis

## Abstract

The complete mitochondrial genome of *Amblyomma geoemydae* is reported for the first time in this study. Its entire mitogenome is 14,780 bp in length, contained 13 protein-coding genes, two ribosomal RNA genes, 22 transfer RNA genes, and two non-coding regions. The phylogenetic analysis by Maximum-likelihood method show that *A. geoemydae* and the others of genus *Amblyomma* are in the same clade, indicating that *A. geoemydae* belongs to the genus *Amblyomma*.

Ticks (Chelicerata: Anactinotrichida: Ixodida) are blood-feeding ectoparasites of terrestrial vertebrates (Burger et al. 2013). *Amblyomma geoemydae* (Ixodida: Ixodidae) is a reptile-associated tick species widely distributed the Southern part of Asia, ranging from South India to the Philippines, Australia and Japan (Bilbija et al. 2019). In China, it is mainly distributed in Taiwan, Jiangsu, Hunan, Hainan, and Guangxi (Sun et al. 2016). The polyphyly of the genus *Amblyomma* is relatively complex (Burger et al. 2013). In this study, the complete mitochondrial genome sequence of *A. geoemydae* was obtained, which provided a basis for the classification study of the genus Amblyomma.

The adult of *A. geoemydae* were collected from the bodies of a chelonian, Nanning City, Guangxi Province, China, on 18 September 2018. The individual tick was stored in the Department of Parasitology, Heilongjiang Bayi Agricultural University (specimen no. BYNKPL-180918). Species identification was conducted by professor Sun Yi based on morphological features. Primers were designed for polymerase chain reaction (PCR) amplification and sequencing on the basis of the mitogenome sequence of *A. cajennense* (GenBank accession no. NC_020333) (Burger et al. 2013).

The total length of *A. geoemydae* mt genome was 14,780 bp (GenBank accession no. MK814531), which contained 13 protein-coding genes (*cox*1-3, *nad*1-6, *nad*4L, *atp*6, *atp*8, and *cyt*b), two rRNA genes, 22 tRNA genes, and two NCR. The arrangement of the *A. geoemydae* was identical with that of hard ticks (Burger et al. 2013; Chang et al. 2019). The *A. geoemydae* mt genome encoded 3614 amino acids in total. The concatenated amino acid sequences of 13 protein-coding genes were analyzed with Maximum-likelihood (ML), using *Nuttalliellidae namaqua* (NC_019663) as out group.

The result show that the tree divided into two large branches: Prostriata and Metastriata. Phylogenetic analysis revealed that the *A. geoemydae* and the others of genus *Amblyomma* are in the same clade (Figure 1). This result supports traditional taxonomic assignment of *A. geoemydae* to the genus *Amblyomma*.

**Figure 1.**
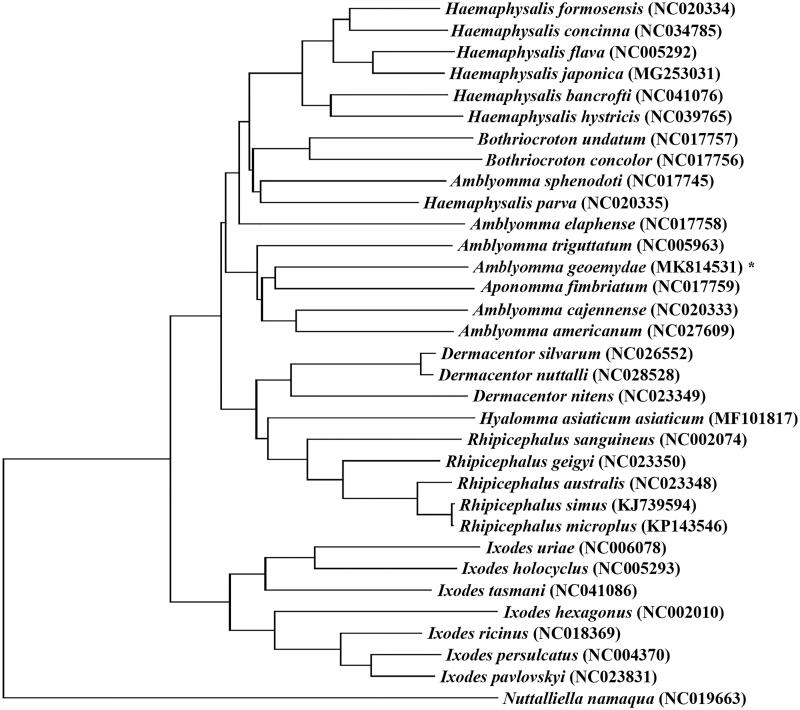
Phylogenetic relationships of *Amblyomma geoemydae* and other species based on mitochondrial sequence data.
